# Scheduling for Emergency Tasks in Industrial Wireless Sensor Networks

**DOI:** 10.3390/s17071674

**Published:** 2017-07-20

**Authors:** Changqing Xia, Xi Jin, Linghe Kong, Peng Zeng

**Affiliations:** 1 Laboratory of Networked Control Systems, Shenyang Institute of Automation, Chinese Academy of Sciences, Shenyang 110016, China; xiachangqing@sia.cn (C.X.); jinxi@sia.cn (X.J.); linghe.kong@sjtu.edu.cn (L.K.); 2 Department of Computer Science and Engineering, Shanghai Jiao Tong University, Shanghai 200240, China

**Keywords:** sensor networks, scheduling, emergency, RM

## Abstract

Wireless sensor networks (WSNs) are widely applied in industrial manufacturing systems. By means of centralized control, the real-time requirement and reliability can be provided by WSNs in industrial production. Furthermore, many approaches reserve resources for situations in which the controller cannot perform centralized resource allocation. The controller assigns these resources as it becomes aware of when and where accidents have occurred. However, the reserved resources are limited, and such incidents are low-probability events. In addition, resource reservation may not be effective since the controller does not know when and where accidents will actually occur. To address this issue, we improve the reliability of scheduling for emergency tasks by proposing a method based on a stealing mechanism. In our method, an emergency task is transmitted by stealing resources allocated to regular flows. The challenges addressed in our work are as follows: (1) emergencies occur only occasionally, but the industrial system must deliver the corresponding flows within their deadlines when they occur; (2) we wish to minimize the impact of emergency flows by reducing the number of stolen flows. The contributions of this work are two-fold: (1) we first define intersections and blocking as new characteristics of flows; and (2) we propose a series of distributed routing algorithms to improve the schedulability and to reduce the impact of emergency flows. We demonstrate that our scheduling algorithm and analysis approach are better than the existing ones by extensive simulations.

## 1. Introduction

Wireless sensor network (WSN) technology is widely applied in process industries because of its advantages of low cost and ease of use [1]. Industrial standards such as WirelessHART [2], WIA-PA [3] and ISA 100.11a [4] have been developed to facilitate the popularization of wireless technology. System reliability and real-time performance are two important indicators for industrial networks. Industrial WSNs must satisfy certain requirements regarding the systems’ real-time performance and reliability when they are used in industrial systems as the communication media [5,6]. To achieve the requirements of high real-time performance and reliability, industrial networks (such as vehicle production line, port logistics dispatching) adopt centralized control mechanisms and allocate resources (slot and channel) before the system begins operation.

**Figure 1:**
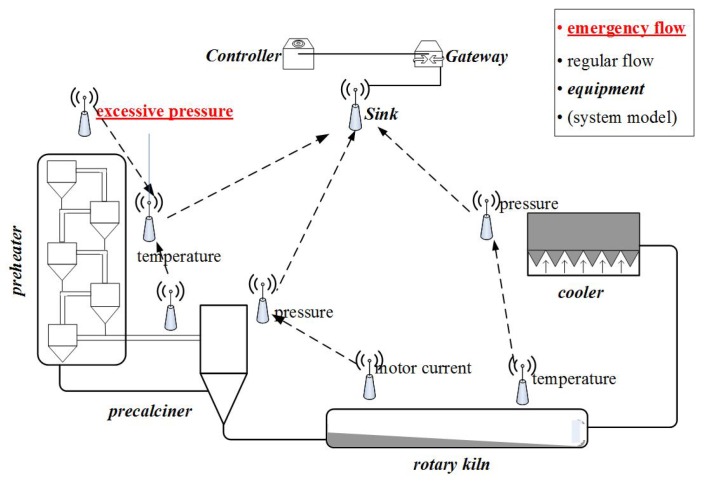
An example of an emergency.

Various kinds of flows co-exist in industrial networks. Some flows have long periods or occur only occasionally, e.g., alarms, accidents and emergencies [7]. The existing methods typically allocate resources without distinguishing or reserving resources for these flows [8]. However, such an approach cannot guarantee the schedulability of emergency flows and consequently leads to a waste of resources. For example, Figure 1 shows an industrial network for the manufacturing of cement; the sensor nodes transmit wirelessly (dotted lines), and the other devices (sink node, gateway and controller) transmit by wired connection (solid line). When an accident occurs, such as excessive pressure, we must forward it to the sink node before its deadline. Sensor nodes are deployed around the factory and monitor each piece of equipment. When an emergency occurs at node $V_{2}$, the emergency packet must reach the controller before its deadline or the quality of the cement will decline. Intuitively, a good result can be achieved if we transmit the emergency task immediately and abandon all other flows when an accident occurs. However, before an emergency actually occurs, nobody knows when and where it will happen. To achieve immediate priority for emergencies, all nodes in the network would need to listen at all times for occasional accidents. In addition, the controller would be required to allocate paths for all possible accident nodes. Obviously, this is impractical in a WSN for the following reasons: (1) the network has limited channels and transmission resources, and it must still satisfy the performance requirements under no-accident conditions; (2) it is not desirable to abandon all regular flows since they also serve useful purposes in the factory; and (3) wireless sensor nodes cannot afford high energy overheads. Hence, it is not only meaningful, but also very urgent to study how to cope with emergencies or accidents in industrial WSNs.

This work systematically investigates the handling of occasional emergency tasks in industrial WSNs, which is a critical issue in many applications, but has thus far received little attention in the existing work. The main challenges are as follows: (1) emergencies occur only occasionally, but the industrial system must deliver the corresponding flows within their deadlines when they occur; and (2) we wish to minimize the impact of emergency flows by reducing the number of stolen flows. In this study, we propose a series of distributed methods that address both the schedulability and impact of flows triggered by emergencies or unpredictable accidents. We list our contributions as follows:

1. We propose an optimal algorithm for emergency flows (optimal path scheduling algorithm (OPSA)); by iterating over all scenarios, the controller can determine the optimal path for an emergency flow. However, because the network is centrally controlled, the source node of the emergency flow needs to upload its information and wait for a response from the network controller. The real-time performance of OPSA is poor. Then, we propose a series of algorithms (such as stealing-first scheduling algorithm (SfSA), lazy stealing scheduling algorithm (LSSA)) to try to improve system performance.
2. We define intersection (such as the intersection nodes or overlap nodes of several flows) and blocking (such as the delay caused by insufficient transmission resources) as new characteristics of flows. The definitions of intersection and blocking will be introduced later.
3. We prove that the shortest path is not always the fastest path for an emergency flow triggered by an unpredictable accident.
4. We analyze the transmission time from $B_{0}$ to the intersection node on the path of flow *i* and obtain the optimal path as the one that satisfies Equation (10) with the minimum number of intersections in $P_{is}$.
5. We propose an algorithm (optimal benefit-based stealing scheduling algorithm (OBSSA)) that can determine optimal paths for emergency flows. The optimal path in the current study is the path that allows an emergency task to arrive at its destination before its deadline with the minimal impact. Furthermore, we prove that the performance of OBSSA is no less than OPSA when we do not consider the overhead of uploading and downloading.

The remainder of the article is organized as follows. The related works are discussed in Section 2. Section 3 presents the system model of an industrial WSN. Section 4 studies scheduling methods for handling unpredictable accidents and proposes several heuristic algorithms. OBSSA is proposed in Section 5. The evaluation of our approaches is presented in Section 6. Section 7 is the conclusion.

## 2. Related Works

Real-time performance and reliability are two critical indicators for industrial WSNs. The algorithms of scheduling and analysis methods of schedulability have been widely studied with respect to these indicators [9,10,11,12]. The work in [13] studies the characteristics of WirelessHART and formulates a real-time transmission scheduling problem by end-to-end analysis. Furthermore, they also prove its NP-hardness. The work in [14] maps real-time periodic flows scheduling in a WirelessHART network to the scheduling of real-time multi-cores. Based on the above two works, [15] reviews recent advances on two fronts: (1) real-time scheduling and analytical methods for obtaining real-time performance in industrial wireless sensor and actuator networks (WSANs); and (2) the CPS (cyber-physical system) co-design of wireless control systems. Haibo Zhang et al. [16] investigated the link-scheduling problem for TDMA-based convergecast, with the aim of minimizing the time consumption of convergecast operation. The work in [17] improves the reliability by means of graph routing. However, none these works considers the differences in the level of criticality between flows. When an emergency flow occurs, it will be treated as a regular flow and miss its deadline.

To improve the reliability of industrial networks, several methods to address critical data flows have been proposed. The work presented in [18] improves the performance for critical data flows by the algorithms of slot assignment, and the work reported in [19] presents similar methods. With the introduction of the concept of a mixed-criticality system [20,21], the schedulability of highly critical data flows in industrial networks can be improved by switching the system into high-criticality mode. Xi Jin et al. [22] proposed an end-to-end delay analysis approach for a mixed-criticality industrial network based on the fixed-priority policy. The work in [8] proposes a scheduling algorithm for a mixed-critical industrial system that guarantees the satisfaction of reliability requirements and real-time performance. Although these previous works differentiate among flows with different critical levels, they do not consider the possibility of accidents or emergencies occurring in the system. Furthermore, we must consider the overhead of system switching and the influence of giving many low criticality flows. The work in [7] presents the first systematic method of incorporating emergency alarms into WPC (wireless process control). Based on event-based communication and slot stealing, the authors of this work propose efficient real-time predictable emergency communication protocols. Building on this work, Jing Li et al. [23] present the RTWS (real-time work stealing) platform, an extension to the widely-applied Cilk Plus concurrency platform. RTWS is mainly used to improve the performance of soft real-time systems. Since the scheduling in the wireless network is different from that in multi-cores, RTWS does not apply to our system (hard real-time industrial network system). However, these works do not consider the possibility of unpredictable accidents or emergencies in the system. That is, there is no corresponding response for unpredictable accidents.

Therefore, existing works cannot cope with unpredictable accidents or emergencies. When these kinds of flows miss their deadline, the disaster of error must occur. To improve the reliability of industrial WSNs and avoid unexpected disasters, it is urgently necessary to develop a reliable scheduling method to resolve this issue.

## 3. System Model

Reliability and real-time performance are the most important criteria for industrial networks [24,25]. When an accident occurs in a physical plant, such as a person entering a dangerous zone, the system must stop the corresponding machine by sending an emergency task to ensure that person’s safety. In such a situation, the most critical concern is to guarantee the timely transmission of the emergency task to the centralized controller. Hence, we consider an industrial system model as follows. The model consists of a gateway, a centralized controller and an industrial WSN (as shown in Figure 1). Based on state-of-the-art industrial network standards [2,3,26], our design has the following salient features: (1) a limited network size; (2) a MAC layer running a multi-channel TDMA protocol; and (3) an IEEE 802.15.4 physical layer that allows per-time-slot channel hopping.

In our design, the sensors constitute a multi-hop wireless mesh network by communicating through a multi-hop WSN. The sensory data are forwarded to the centralized controller as their destination. The set of sensor nodes is denoted by $N=\{n_{1},n_{2}\ldots \}$. The sink node $N_{sink}$ is the destination for all flows. There are 16 non-overlapping channels in our physical layer, which is the same as the IEEE 802.15.4 protocol. The number of channels in our model is denoted by *M* and satisfies 1≤M≤16 [27].

The flow in our model is defined as an end-to-end communication between a source and a destination. There are two kinds of data flows in our model: periodic regular flows and aperiodic emergency flows (for naming consistency, we refer to an occasional emergency task as an emergency flow). For regular flows, packets are generated periodically. Emergency flows are triggered sporadically. An emergency flow is more important than a regular flow. We take gas monitoring as an example: the regular flows are $CO_{2}$, $O_{2}$ and the emergency flow is CO. When there are CO leakages, the system must forward the information of the toxic leakage to its deadline in time, or a disaster may occur. We consider a flow set that contains *r* regular flows and *e* emergency flows, which is denoted by $F=\{R_{1},R_{2}\ldots R_{r},E_{1},E_{2}\ldots E_{e}\}$. The characteristics of a flow in our system are denoted by {c,d,t,p}, where *c* is the number of transmission hops (the number of hops in this paper is the number of links from the source node to its destination); *d* is the packet deadline (*d* is also the packet arrival time when all slots are assigned by the network controller); *t* is the period of the flow; and *p* is the path, which stores the sensor nodes through which the packet is transmitted. Hence, for any flow *i*, we can describe it as follows. The flow generates packets with a period of $t_{i}$, via $c_{i}$ hops on the routing path $p_{i}$, and it forwards each packet to its destination before $d_{i}$.

We define an emergency flow as a predictable emergency flow when it is generated at a particular location and transmitted over a prescribed path (such as alarms or the other devices for which errors or accidents may occur). The characteristics of predictable emergency flow are {C,D,P}, where *C* is the number of transmission hops, *D* is the deadline of the emergency flow and *P* is the path; otherwise, the emergency flow is unpredictable and could be generated with the source node of any regular flow as its source node. Hence, we let *A* denote the source of such an emergency flow, and we let *B* denote the next hop of that emergency flow. Then, an unpredictable emergency flow can be characterized by {A,D}. To clarify the actual execution time, we use *C* to denote the actual number of transmission hops from the source to the destination. Since the packet can be transmitted by one hop in each transmission slot, we can use the actual number of transmission hops *C* to represent the transmission time, and the unit of *C* is the slot. For both predictable and unpredictable emergency flows, such a flow can be scheduled when the number of slots required for the transmission of the emergency flow to its destination is no more than *D*.

Previous research has mainly focused on predictable emergency flows, and the most common approach is to reserve or pre-allocate resources for these flows. However, this method is only applicable to predictable emergency flows. In addition, the probability of these emergency flows is very low; consequently, the resources allocated to predictable emergency flows are wasted when no emergency flows are triggered. To achieve both the consideration of unpredictable emergency flows and more efficient utilization of resources (channels and slots), we introduce a stealing mechanism. In this stealing mechanism, the centralized controller allocates resources to sensor nodes only for regular flows under the assumption that there are no emergency flows. The resources considered are as follows.

1. In which time slots a sensor node can send and receive messages.
2. Which channel can be used when a sensor node sends a message.
3. Which channel can be used when a sensor node receives a message.
4. Which flow a transmitted message belongs to and the location of the current node on that flow’s path.
5. When a message transmitted by a node can reach its destination.

**Figure 2:**
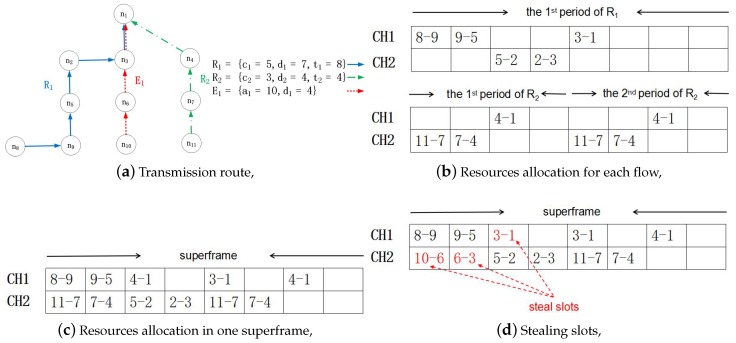
An example of the stealing mechanism.

A superframe consists of schedules with the same period [27], and the deadline of any packet that is generated by a regular flow that must lie within a superframe. That is, the flow can be scheduled only when its packet can reach the destination before its deadline. Hence, superframes repeat periodically, allowing regular flows to be transmitted successfully (the length of the superframe in our model is the lowest common multiple of the periods of all regular flows and is denoted by *T*). However, this scenario holds only when only regular flows are present in the network. When an emergency flow is triggered, it must be transmitted to the destination before its deadline. Then, the stealing mechanism comes into effect. A packet that is generated by an emergency flow can steal resources allocated to regular flows, as shown in Figure 2.

There are two regular flows and one emergency flow in this example, with the flow characteristics in Figure 2a–c, showing the resource allocation for regular flows. CH is the abbreviation of channel, and there are two channels (CH1 and CH2). Since no resources have been allocated for the emergency flow, it can steal slots that are allocated to regular flows to ensure its schedulability. Hence, as shown in Figure 2d, $E_{1}$ steals three slots. This stealing mechanism causes $R_{2}$ to miss its deadline in the first period.

From this example, it is not difficult to see that it is very important to develop a reasonable method by which emergency flows can steal resources. Since almost all of the data in the industrial system are useful (for example, temperature is not a critical parameter in many situations; however, it has a great effect in some accidents playing the role of assistant analysis). An unreasonable stealing method may cause the system’s acceptance ratio to significantly decline. If $E_{1}$ were to use CH2 in Time Slot 3, both $R_{1}$ and $R_{2}$ would be discarded. Obviously, we want the system to have a high acceptance ratio.

A rate-monotonic (RM) scheduling scheme is the state-of-the-art technique for the allocation of flow priority in real-time systems. A flow’s priority is inversely proportional to its period. Thus, for flows *i* and *j*, the priority of flow *j* is higher than that of flow *i* when $t_{i}>t_{j}$. As stated in our previous work, although an emergency flow does not have a period, we allocate the highest priority to emergency flows when they are present.

In the industrial system, we attach great importance to the schedulability of the system (whether the system is schedulable or not). Then, the concept of a schedulable flow set is defined as follows. When only regular flows exist in the network, the flow set is schedulable if all flows can meet their deadlines. When an emergency flow is present, the flow set is schedulable if all emergency flows can meet their deadlines. Since emergencies occur only occasionally, we assume that at most, one emergency flow will simultaneously exist in the network. Furthermore, our work is a kind of scheduling method to improve the schedulability of emergency flow based on the existing network; hence, we do not consider the deployment and connectivity (these issue should be considered in the existing network design phase).

## 4. Scheduling for Unpredictable Accidents

We describe the maximum acceptance rate scheduling problem based on the above system model as follows. Given the flow set F and the network, our objective is to schedule transmissions with the limited network resources (time slot and channel) such that the flow set can be scheduled. In addition, when an emergency flow is triggered, we want to maximize the acceptance ratio for regular flows under the condition that the emergency flow is schedulable. As Figure 2 shows, the emergency flow can be forwarded to its destination with the minimal impact by means of a suitable stealing mechanism.

In this section, we first formulate the problem. Then, we propose our optimal algorithms for reducing the impact of emergency flows.

### 4.1. Problem Statement

Before the system begins operation, the controller will allocate resources for each regular flow. Nodes in the network forward packets via multi-hop transmission. When the resources are not enough, the flow with low priority must be delayed or even miss its deadline. Given a network routing, two kinds of delays might be generated in the system, which can be summarized as follows:

1. Channel contention: Across the entire network, each channel can only be allocated to one transmission in the same slot.
2. Transmission conflicts: Each node can perform only reception or transmission of one packet in each slot. Hence, when two transmissions conflict, the transmission that belongs to the lower priority flow must be delayed for the higher priority one.

For regular flows, their transmission resources are allocated by the network controller. Each regular flow is allocated an RM scheme. If several flows have the same RM priority, the controller will assign priorities by the flow’s ID. The priority of a transmission is equal to the priority of its flow. The controller sends these allocations to each node. The set of slots for packets sent by node *k* in one superframe is denoted by $W^{k}=\left\{w{\left(i,j\right)}^{k}\right\}$, where *i* is the flow ID and *j* is the transmission index. Hence, $w{\left(i,j\right)}^{k}$ is the slot in which the *j*-th transmission on flow *i* is sent from node *k*. Let us present an illustrative example based on Figure 2. For $R_{2}$, the second time for which Node 7 sends a packet on $R_{2}$ is in the sixth slot; hence, $w{\left(2,2\right)}^{7}=6$. $W^{k}$ is arranged in ascending order, and initially, j=0; the set of slots for packet forwards from node *g* to the destination in one superframe is denoted by $R^{g}=\left\{r{\left(i,j\right)}^{g}\right\}$. The time slot in which packet *j* generated by flow *i* reaches the destination is denoted by r(i,j). We also illustrate this by Figure 2. For $R_{2}$, the second time for which Node 4 receives a packet generated by $R_{2}$ is in the sixth slot; hence, $r{\left(2,2\right)}^{4}=6$. The set of nodes on flow *i* is denoted by $H_{i}=\left\{h\left(i,q\right)\right\}$, and k=h(i,q), where *i* is the flow ID and *k* is the *q*-th node on flow *i*. Then, the location of node *k* on the path of flow *i* can be obtained when k=h(i,q); if $k\notin H_{i}$, then node *k* is not on the path of flow *i*. In the previous example based on Figure 2, both Node 7 and Node 4 are on $R_{2}$; hence, h(2,1)=7 and h(2,2)=4.

Since no resources are allocated for emergency flows, when an emergency flow is triggered, it must steal resources allocated for regular flows. Any regular flow from which resources are stolen must miss its deadline. In addition, although the emergency flow has the highest priority, it cannot steal resources arbitrarily. The stealing behavior results only in the takeover of previously-allocated time slots or the corresponding receiving node might miss receiving the packet, which would cause the emergency flow to miss its deadline. We let *S* denote the number of stolen flows; then, our objective is min{S} with limited resources.

(1) $$\begin{array}{c} \min\{S\},\\ s.t.\;C\leq D. \end{array}$$

### 4.2. Optimal Path Scheduling Algorithm

In this subsection, we propose the OPSA for a conventional centralized network. The key idea is that by iterating over all scenarios, the controller determines the optimal path for an emergency flow. We characterize the path by {speed,S}, where speed is the transmission time from the source node of the emergency flow to the destination and *S* is the number of stolen flows. Emergency flow *E* is generated at node *A* (A∈N) and is released in time slot Slot (Slot<T). The controller can always determine the optimal path by traversing all possible scenarios, and then, the controller sends this information to each node. Regardless of where and when the accident occurs, the emergency flow can always be transmitted on the optimal path.

The pseudo-code for OPSA is shown in Algorithm 1. When an emergency occurs at node *A*, we initialize *S* and speed and upload the information concerning the emergency to the controller (Lines 1–4). Then, the controller searches for a path and updates {speed,S} through iteration (Lines 5–10), where *r* is the number of regular flows. The optimal path is obtained by comparing *S* for each path that satisfies speed≤D and returning the {speed,S} that satisfies min{S}. Then, the emergency flow is transmitted on the optimal path (Lines 11–15).

The time complexity of OPSA is $O(r^{2})$. However, because the network is controlled in a centralized manner, the source node of the emergency flow needs to upload its information and wait for a response from the network controller. Obviously, the real-time performance of OPSA is poor.

**Algorithm 1** Optimal path scheduling algorithm.**Input:** the flow set *F*; **Output:** the optimal path, {speed,S};
  1:an emergency occurs at node *A*;  2:S←0;  3:speed←0;  4:upload emergency information to the controller;  5:**for** i=1 to *r* **do**  6: **for** j=1 to *r* **do**  7:  determine the number of paths *n* from the source node *A* to the destination;  8:  update {speed,S} for each path;  9: **end for**10:**end for**11:**for** i=1 to *n* **do**12: find the optimal path that satisfies min{S} and speed≤D;13: **return** the optimal path and the corresponding {speed,S};14:**end for**15:download the optimal path to node *A*, then transmit the emergency flow on this path;


To address this issue, we consider a series of approaches to achieving distributed path selection with an acceptable time complexity and real-time performance. The objective is to select a path for the emergency flow that guarantees the network’s schedulability with a lower time complexity and higher real-time performance than OPSA. To achieve this goal, we choose a path for the emergency flow in a distributed manner and make decisions based on the information available at the current node.

### 4.3. Stealing-First Scheduling Algorithm

In this subsection, we propose a trivial algorithm called the stealing-first scheduling algorithm (SfSA). In SfSA, the emergency flow has the highest priority and can steal all resources from regular flows at the current node. The emergency flow obeys a first-fit stealing mechanism, meaning that the emergency flow will steal resources in the next time slot.

The pseudo-code for SfSA is shown in Algorithm 2. Before the algorithm starts, we input the number of channels, *M*; the flow set, *F*; the information of each node; and the characteristics of the emergency flow. The outputs are the schedulability of the emergency flow and the number of stolen flows, *S*. When an emergency flow is triggered, the arrival time and the number of stolen flows *S* are stored (Lines 1–3). Then, the emergency flow packet is transmitted from the source node *A* to the destination based on a first-fit sending slot principle (Lines 4–14). Node *A* checks its set of sending slots and finds the first sending slot that satisfies w(i,j)≥Slot, where Slot is the current time slot, and then calculates the remaining time D−(w(i,j)−Slot) (Lines 5–6). If the remaining time is less than zero, the emergency flow will miss its deadline, and a value of ‘unscheduled’ is returned; otherwise, the next-hop node for which *A* is scheduled to transmit in time slot w(i,j) becomes the next target of the emergency flow, which means that the slot is stolen from the original flow. Hence, the target node $h_{i}$ updates its source in the next loop, and the number of stolen flows is incremented by one (Lines 7–13). When the packet generated by the emergency flow reaches its destination, the system returns the total number of stolen flows (Lines 14–15).

**Algorithm 2** Stealing-first scheduling algorithm.**Input:** the flow set *F*; each node’s information, $W^{g}$ and $R^{g}$ for g∈N and $H_{i}$ for i∈F; the deadline *D* of the emergency flow and its source *A*; **Output:** the schedulability of the emergency flow and the acceptance ratio for regular flows;
  1:Query the sending slots and next-hop destinations at node *A*;  2:Slot← the arrival time of the emergency flow;  3:S←0;  4:**while** (A!=destination) **do**  5: w(i,j)← the first transmission slot in $W_{A}$ that satisfies w(i,j)≥Slot at node *A*;  6: D←D−(w(i,j)−Slot);  7: **if** D<0 **then**  8:  **return** unscheduled;  9:  break;10: **else**11:  $A\leftarrow h(i,q_{i}+1)$;12: **end if**13: S←S+1;14:**end while**15:**return** *S*;

The number of iterations of the while loop beginning at Line 4 is O(n); hence, the time complexity of Algorithm 2 is O(n).

### 4.4. Lazy Stealing Scheduling Algorithm

**Figure 3:**
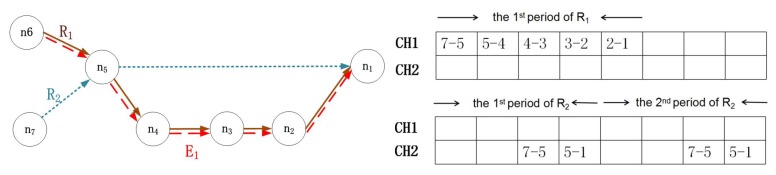
An example of stealing-first scheduling algorithm (SfSA).

For the consideration of emergency situations, we proposed SfSA in the previous subsection. However, this algorithm may result in a low acceptance ratio for regular flows. In addition, the first-fit sending slot principle cannot guarantee the schedulability of an emergency flow, when the first available choice does not provide the most direct path. We present an example of SfSA based on Figure 3. The characteristics of emergency flow $E_{1}$ are $\left\{a_{1}=6,d_{1}=4\right\}$. Then, $E_{1}$ will transmit on the path of $R_{1}$ based on the first fit policy, and the transmission time is five. Since 5>4, $E_{1}$ will miss its deadline. Hence, in this subsection, we propose the lazy stealing scheduling algorithm (LSSA) with the objective of enhancing both the schedulability and the acceptance ratio. Several rules are imposed in LSSA:

1. When seeking schedulability, the emergency flow prioritizes stealing resources from a flow from which resources have already been stolen.
2. The emergency flow stops stealing once it becomes schedulable.
3. The emergency flow prioritizes stealing resources from the most suitable flow. We define the most suitable flow as the one that satisfies:
  (2) $$\min\{\Delta_{i}+\left(w{\left(i,j\right)}^{B}-t_{current}\right)\},i\in F,$$
  where $\Delta_{i}$ is the distance from the current node to the destination on the path of flow *i*, $t_{current}$ is the current time slot and *B* is the current node.
4. When the distance from the current node to the destination is the same for several flows, the emergency flow prioritizes stealing from the flow that is scheduled for the first transmission. We evaluate the distance by comparing the number of hops from the current node to the destination.

By means of the first two rules, we increase the network acceptance ratio by reducing unnecessary stealing. The system stores the set of flows from which resources have been stolen as Θ. When an emergency flow can be scheduled, it will stop stealing and transmit on its current path; otherwise, it will prioritize choosing a path in Θ.

According to the third rule, the emergency flow will prioritize stealing the flow that is most suitable rather than the one that is the first to be transmitted. In LSSA, suitability is a relative value, which means that the emergency flow will choose the path that is quickest and nearest to the destination at the current node. Finally, if there are several paths with the same distance, the emergency flow will steal resources from the flow that is to be sent first according to $W_{i}$. Obviously, the emergency flow will arrive at its destination when $\Delta_{i}$ is equal to zero.

The pseudo-code for LSSA is shown in Algorithm 3. The inputs and outputs are the same as for SfSA. When an emergency flow *E* is triggered at an arbitrary node *A*, node *A* queries the relevant information and initializes the stolen flow set Θ (Lines 1–3). The stealing mechanism in LSSA follows the rules presented above. First, it is determined whether the emergency flow *E* can be scheduled. If *E* can be scheduled, it will stop stealing, and a value of ‘scheduled’ will be returned (Lines 4–7). If not, it is determined whether there is a flow in Θ that satisfies $d_{i}\leq D$. If so, then flow *E* takes the place of this flow (Lines 8–10). If none of the flows in Θ satisfies $d_{i}\leq D$, then Equation (2) is used to select a target flow from which resources have not already been stolen (Lines 11–12). When there are several paths with the same suitability, the one that is scheduled for transmission in the earliest slot is selected (Lines 13–18). When the emergency flow arrives at its destination, the algorithm returns the number of flows from which resources have been stolen. If no flow satisfies $d_{i}\leq D$, then a value of ‘unscheduled’ is returned (Lines 19–27). The time complexity of Algorithm 3 is O(n).

**Algorithm 3** Lazy stealing scheduling algorithm.**Input:** the flow set *F*; each node’s information, $W^{g}$ and $R^{g}$ for g∈N and $H_{i}$ for i∈F; the deadline *D* of the emergency flow and its source *A*; **Output:** the schedulability of the emergency flow and the acceptance ratio for regular flows;
  1:Query the information of node *A*;  2:Slot← the arrival time of the emergency flow;  3:Θ←∅  4:**while** (A!=destination) **do**  5: **if** the emergency flow can meet its deadline on the current path **then**  6:  **return** scheduled;  7:  break;  8: **else**  9:  **if** $\exists R^{h(i,q_{i}+1)}\in \Theta$ satisfies $d_{i}\leq D$ **then**10:   $A\leftarrow h(i,q_{i}+1)$;11:  **else**12:   i← the path satisfies $\min\{\Delta_{i}+\left(w{\left(i,j\right)}^{B}-t_{current}\right)\},i\in F$;13:   **if** $\exists \left(c_{j}-q_{j}\right)==\left(c_{k}-q_{k}\right)$ **then**14:    i← the next transmission between *j* and *k*;15:    *i* join Θ;16:    $A\leftarrow h(i,q_{i}+1)$;17:   **end if**18:  **end if**19:  Slot++;20:  **if** Slot>D **then**21:   **return** unscheduled;22:   break;23:  **end if**24: **end if**25:**end while**26:S← the length of Θ;27:**return** *S*;

## 5. Optimal Benefit-Based Stealing Scheduling Algorithm

**Figure 4:**
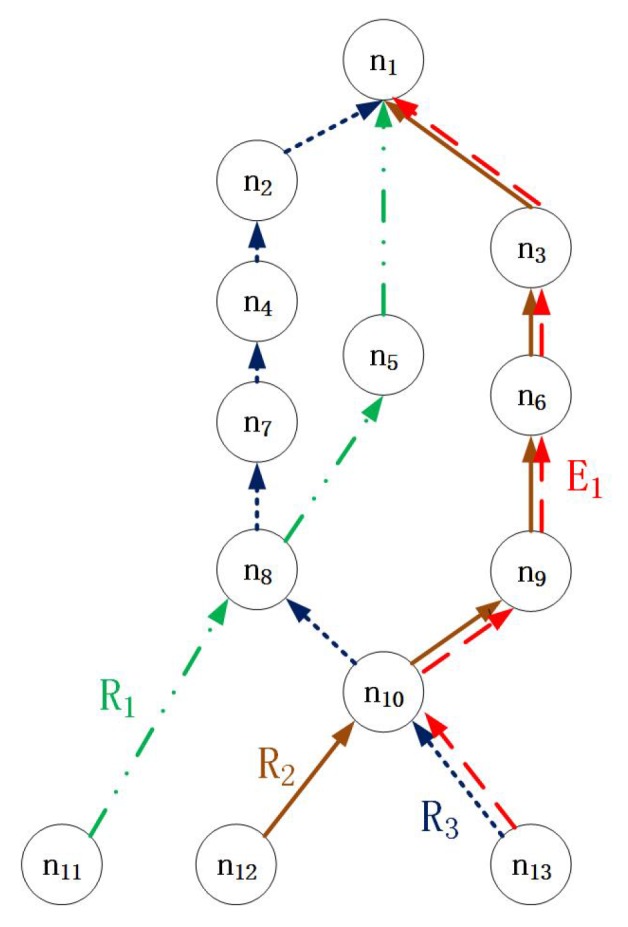
An example of indirect routing.

In LSSA, the flow from which resources will be stolen is chosen using Equation (2). However, this may cause the emergency flow to not take the most direct route when there is a shortcut available on a path that initially appears longer. In Figure 4, when an emergency flow occurs at Node 13, Equation (2) would lead to the selection of the red path; however, the shortest path is Node13→Node10→Node8→Node5→Node1.

To address this issue, we propose the optimal benefit-based stealing scheduling algorithm (OBSSA) to avoid indirect routes and transmission congestion. Through real-time path selection, OBSSA can improve the schedulability of emergency flows.

We introduce OBSSA based on three considerations: (1) we first define intersections and blocking and analyze the characteristics of the network accordingly; (2) based on these network characteristics, we propose a method that can guarantee that an emergency flow will arrive at its destination before its deadline (although our method cannot schedule an emergency flow when there is no path that can transmit the packet to its destination before its deadline); and (3) we design a data structure for the additional memory overhead.

**Definition** **1.** (Intersection) Consider a node k that is on the path of flow $F_{i}$ and is also on the path of flow $F_{j}$. If node k is the start or end of an overlap between the paths of $F_{i}$ and $F_{j}$, then node k is an intersection between $F_{i}$ and $F_{j}$; if node k is not the start or end of such an overlap, it is not an intersection. The intersection information is stored on node k.

**Figure 5:**
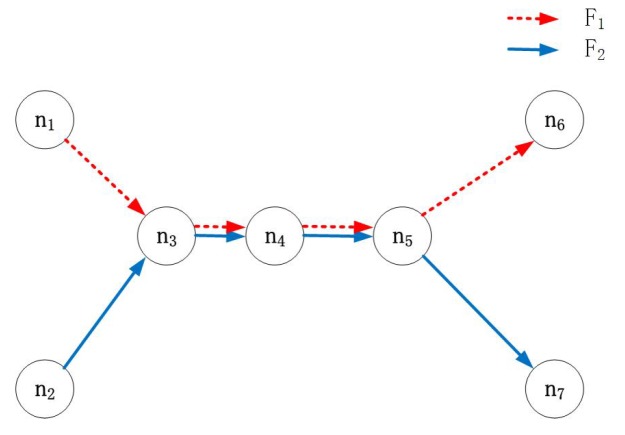
An example of intersections.

In Figure 5, two flows intersect at Node 3–Node 5. The overlap starts and ends at Node 3 and Node 5, respectively; hence, these nodes are intersections between $F_{1}$ and $F_{2}$. However, Node 4 is not an intersection (since Node 4 is neither the start nor the end of the overlap).

We let {λ,ξ} denote the number of intersections on the path of a flow and their locations, where ξ is a node set that stores each intersection’s location. Then, we can extend the set of characteristics of each regular flow to {c,d,t,p,λ,ξ}. For $R_{3}$ in Figure 4, there are two intersections, at the first and second hops on the path, Node 10 and Node 8. Hence, λ=2 and ξ=1,2 in this situation. For simplicity, we assume here that two flows will have at most one intersection; however, our method can be used directly in systems with an arbitrary number of intersections.

The pseudo-code for intersection identification (II) is shown in Algorithm 4. Given the characteristics of each regular flow, we update the intersections using II.

When emergency flow *E* cannot be scheduled on the current path and is transmitted to an intersection, it must make a decision regarding which path to follow. There are *j* choices of possible paths when *j* regular flows crossing at node *B*. Obviously, *E* will prioritize choosing a path that can reach the destination before its deadline. We mainly study the situation in which no path can be clearly confirmed to be capable of forwarding *E* to its destination before its deadline. Since an accident may occur at any source node and in any time slot, we cannot pre-assign an optimal path for an emergency flow. However, the emergency flow may miss its deadline if it selects its path only by choosing the largest λ at node *B*. Thus, we should consider both the remaining time slots and the remaining intersections.

**Algorithm 4** Intersection identification.**Input:** E; **Output:** update the characteristics of each regular flow;
1:**for** each flow $F_{i}$ **do**2: **for** each flow $F_{j}$, i≠j **do**3:  **if** node *k*∈$F_{i}$, node *k*∈$F_{j}$, and node *k* is the start or end of the overlap between $F_{i}$ and $F_{j}$ **then**4:   *k* join $\xi_{i}$;5:   $\lambda_{i}++$;6:  **end if**7: **end for**8:**end for**9:**return** the characteristics of each regular flow;

**Lemma** **1.** *Given a number of input paths* Ω *and a number of output paths ω at intersection node B, Ω≥ω.*

**Proof.**  We prove this lemma by contradiction. The number of input paths Ω at intersection node *B* is the number of input flows, and it is easy to obtain form node B. Suppose that the number of output paths is larger than the number of input flows, that is, Ω<ω. Then, there must be at least one additional path that is useless. However, the controller will not allocate transmission slots for nodes to compose such a path, which contradicts the given condition. Hence, given a number of input paths Ω and a number of output paths ω at intersection node *B*, Ω≥ω. ☐

**Lemma** **2.** Given a network in which time slots are allocated for regular flows, the shortest path is not always the fastest path for an emergency flow triggered by an unpredictable accident.

**Proof.**  We prove this lemma by an example. In Figure 4, the shortest path from Node 13 to the destination, Node 1, is Node13→Node10→Node8→Node5→Node1. The emergency flow $E_{1}$ could arrive at the same time as $R_{3}$ by simply stealing $R_{3}$’s resources instead of taking the shortest path; then, it would reach the destination by $d_{3}$. However, if the period of regular flow $R_{1}$ is longer than that of $R_{3}$ and a packet from $R_{1}$ has just been sent from Node 8 to Node 5, then the transmission of the emergency flow from Node 8 to Node 5 will be blocked for an entire period $t_{1}$. In this case, if the emergency flow takes the shortest path, then $E_{1}$ will ultimately reach the destination after $\left(2+t_{1}+2\right)$ slots, and it is easy to find that $\left(2+t_{1}+2\right)>t_{3}\geq d_{3}$. Hence, the shortest path is not always the fastest path for an emergency flow triggered by an unpredictable accident. ☐

We define the phenomenon of blocking of an emergency flow as follows.

**Definition** **2.** *(Blocking) Blocking is the phenomenon in which an emergency flow cannot be transmitted from the current node to the next-hop destination because there are no sending resources available. For the current node k, the one-hop blocking time on path i is:*

(3) $${ℵ}_{i}^{k}=w{\left(i,j\right)}^{k}-t_{current}.$$

Since we represent the intersection information for a flow as {λ,ξ}, we can use the notation $\xi^{k}$ to express that node *k* is the $\left(\xi^{k}\right)$-th intersection on the path of flow *i*. Then, we can obtain the number of remaining intersections for flow *i*, $\alpha_{i}^{k}$, as follows:

(4) $$\alpha_{i}^{k}=\lambda_{i}-\xi_{i}^{k}.$$

**Figure 6:**
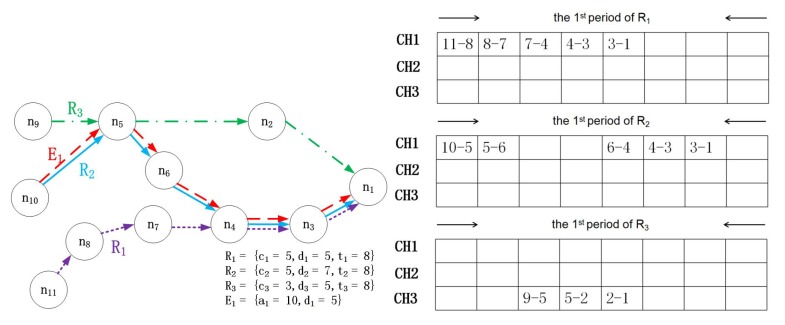
An example for selecting the path based only on blocking and intersections.

Obviously, it is difficult to select an optimal path based only on blocking and intersections. It is not always a good decision for an emergency flow to steal resources from flow *i* when node *k* has the smallest ${ℵ}_{i}^{k}$ and the largest $\alpha_{i}^{k}$ on the path of flow *i*. For example, in Figure 6, the emergency flow must miss its deadline by selecting the path based only on blocking and intersections. Obviously, there exists one path Node10→Node5→Node2→Node1 that can guarantee the schedulability of $E_{1}$. To improve the schedulability of the emergency flow and guarantee that it will be transmitted on an optimal path, we treat intersection nodes as beacon nodes. Each beacon node stores the information of all intersections, which is denoted by {W,H}. Hence, a path can be selected based on a long-term prediction of the end-to-end delay.

When an emergency flow packet is forwarded at intersection node $B_{0}$, we first search for a path that satisfies r(i,j)≤D (find a flow that can meet the emergency flow’s deadline). Then, we evaluate the number of time slots required for the emergency packet to be transmitted from $B_{0}$ to the intersection node on the path of flow *i* ($B_{i}$); this transmission time is denoted by $\varphi (B_{0},i)$. The optimal path can be determined as follows:

(5) $$\varphi \left(B_{0},i\right)+r{\left(i,j\right)}^{B_{i}}\leq D.$$

The optimal path is the one with the minimum number of stolen flows from $B_{0}$ to $B_{i}$. This means that the optimal path can guarantee the emergency flow’s schedulability with the minimum number of stolen flows.

**Figure 7:**
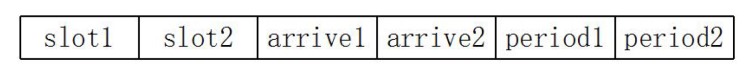
Data structure.

We use a list data structure to store the intersection information, as shown in Figure 7. There are six elements, where slot1 is the first time slot for transmission through the intersection for the flow corresponding to the row; the corresponding arrival time is arrive1; and period1 is the flow’s period. Similarly, slot2, arrive2 and period2 represent the same information for the flow corresponding to the column.

**Theorem** **1.** When an emergency packet is transmitted to an intersection, it can obtain the time slot for the transmission of the next packet to another intersection.

**Proof.**  When an emergency packet is forwarded to an intersection in any time slot $t_{current}$, the node will look for information on other intersections. Then, it can obtain the time slot for the transmission of the next packet to another intersection as follows:

(6) $$\left\{\begin{array}{cc} r\left(i,1\right)-t_{current}\;\;\mathrm{mod}\;t_{i} & r\left(i,1\right)\geq t_{current}\;\;\mathrm{mod}\;t_{i}\\ t_{i}-\left(t_{current}\;\;\mathrm{mod}\;t_{i}-r\left(i,1\right)\right) & r\left(i,1\right)<t_{current}\;\;\mathrm{mod}\;t_{i}. \end{array}\right.$$

When $r\left(i,1\right)\geq t_{current}\;\mathrm{mod}\;t_{i}$, this means that the next packet has not passed the intersection, and the waiting time is $r\left(i,1\right)-t_{current}\;\mathrm{mod}\;t_{i}$; otherwise, the intersection will wait for the packet to be released from the source node, and the next packet will reach it $t_{i}-\left(t_{current}\;\mathrm{mod}\;t_{i}-r\left(i,1\right)\right)$ slots later. Hence, when an emergency packet is transmitted to an intersection, it can obtain the time slot for the transmission of the next packet to another intersection. ☐

**Theorem** **2.** *We let $P_{is}=\left\{B_{0},B_{1},\ldots B_{k}\ldots \right\}$ denote the intersection set for the forwarding of an emergency packet from the current intersection node $B_{0}$ to the destination. Then, the transmission time $\varphi (B_{0},i)$ is:*

(7) $$\varphi \left(B_{0},i\right)=\sum_{k=0}^{i-1}\left({ℵ}^{B_{k}}+t^{B_{k}\to B_{k+1}}\right),B_{k}\in P_{is},$$

*where $t^{B_{k}\to B_{k+1}}$ is the transmission time from $B_{k}$ to $B_{k+1}$. Since $B_{k}$ and $B_{k+1}$ are on the path of the same flow, $t^{B_{k}\to B_{k+1}}$ is easy to obtain.*

**Proof.**  The emergency packet is blocked at $B_{0}$ for ${ℵ}^{B_{0}}$ time slots, and it is then forwarded on the path from node $B_{0}$ to $B_{1}$. Since $B_{0}$ and $B_{1}$ are on the path of the same flow, we can obtain $t^{B_{0}\to B_{1}}$ as:

(8) $$(w{\left(g,j\right)}^{B_{1}}-w{\left(g,j\right)}^{B_{0}}).$$

The transmission time from node $B_{0}$ to $B_{1}$ is:

(9) $$({ℵ}^{B_{0}}+t^{B_{0}\to B_{1}}).$$

The remaining transmission processes are the same as that from $B_{0}$ to $B_{1}$. Hence, we can obtain the transmission time $\varphi (B_{0},i)$. ☐

**Theorem** **3.** *The optimal path is the one that satisfies:*

(10) $$\sum_{k=0}^{i-1}\left({ℵ}^{B_{k}}+t^{B_{k}\to B_{k+1}}\right)+r{\left(i,j\right)}^{B_{i}}\leq D,B_{k}\in P_{is}$$

*with the minimum number of intersections in $P_{is}$. We let ϵ denote the minimum length of $P_{is}$. The number of stolen flows is ϵ+1.*

**Proof.**  If a path satisfies Equation (10), this means that the emergency packet could be scheduled on that path. However, there may be several paths that satisfy Equation (10). We select the path with the minimum number of intersections in $P_{is}$ during the stealing process. The emergency packet changes flow paths at each intersection. Hence, the number of stolen flows is ϵ+1. ☐

We propose OBSSA to select the path for an emergency flow based on Equation (10), and the pseudo-code for OBSSA is shown in Algorithm 5.

**Theorem** **4.** The performance of OBSSA is no less than OPSA even though the overhead of the upload and download in OPSA is not taken into account.

**Proof.**  We prove this theorem by contradiction. Then controller generates the control flow at the beginning of superframe. Hence, the response time of upload and download in OPSA is much bigger than the emergency flow’s deadline. To analyze the performance of OBSSA, we do not consider the unacceptable overhead in OPSA. When the emergency flow can be scheduled by OPSA, there is at least one optimal path that allows the emergency flow to arrive at its destination before its deadline with the minimal impact (*S*). If the performance of OBSSA is less than OPSA, OBSSA cannot guarantee the schedulability or the number of stolen flows in OBSSA is more than in OPSA, which contradicts Equation (1) and Theorem 3. Hence, the performance of OBSSA is no less than OPSA even though the overhead of the upload and download in OPSA is not taken into account. ☐

**Algorithm 5** Optimal benefit-based stealing scheduling algorithm.**Input:** the flow set *F*; each node’s information, $W^{g}$ and $R^{g}$ for g∈N and $H_{i}$ for i∈F; the deadline *D* of the emergency flow and its source *A*; **Output:** the schedulability of the emergency flow and the acceptance ratio for regular flows;
  1:query the information of node *A*;  2:Slot← the arrival time of the emergency flow;  3:Θ←∅  4:**while** (A!=destination) **do**  5: **if** the emergency flow can meet its deadline on the current path **then**  6:  **return** scheduled;  7:  break;  8: **else**  9:  **if** $\exists R^{h(i,q_{i}+1)}\in \Theta$ satisfies $d_{i}\leq D$ **then**10:   $A\leftarrow h(i,q_{i}+1)$;11:  **else**12:   i← the path that satisfies $\min\{a_{i}\frac{{ℵ}_{i}^{k}}{\alpha_{i}^{k}-1}\},i\in F$;13:   **if** $\exists \left(c_{j}-q_{j}\right)==\left(c_{k}-q_{k}\right)$ **then**14:    i← the next transmission between *j* and *k*;15:    *i* join Θ;16:    $A\leftarrow h(i,q_{i}+1)$;17:   **end if**18:  **end if**19:  Slot++;20:  **if** Slot>D **then**21:   **return** unscheduled;22:   break;23:  **end if**24: **end if**25:**end while**26:S← the length of Θ;27:**return** *S*;


### Complexity Analysis

In this subsection, we analyze the complexity of OBSSA. Obviously, the time complexity is O(n). Regarding the space complexity, we store the information on each intersection using an *F*-order matrix *M*. The numbers of rows and columns of the matrix is equal to the number of flows. $M_{ij}=0$ if $F_{i}$ and $F_{j}$ are disjoint with each other; otherwise, $M_{ij}$ is the ID of the intersection node. The information on $F_{i}$ and $F_{j}$ is also stored in $M_{ij}$, such as the first transmission time slot and the period.

Since any two flows have at most one intersection, the maximum number of intersections is:

(11) $$\frac{F(F-1)}{2}.$$

Since the length of the data structure is six, the total number of elements is:

(12) $$\frac{6F(F-1)}{2}=3F\left(F-1\right).$$

Hence, the space complexity of storing all intersection information is $S\left(F^{2}\right)=3F\left(F-1\right)$. In other words, we improve the scheduling performance by increasing the space complexity by an acceptable amount. Considering the typical size of an industrial network (usually fewer than 100 nodes) [13,27], the additional space required to store the intersection information represents an acceptable increase in space complexity even when the number of intersections is unconstrained.

## 6. Experiment

In this section, we present experiments conducted to evaluate the performance of our proposed methods. Since there is no similar previous research about stealing mechanisms in industrial WSNs, we compare our methods (OBSSA and SfSA) with the traditional RM scheme without stealing [28] and with the shortest path Dijkstra algorithm [29] in terms of both the schedulability ratio and the number of stolen flows. The schedulability ratio can be obtained by $\frac{z}{Z}$, where *Z* is the number of executions, and *z* is the number of success executions. Since OPSA has a long response time, we do not consider OPSA in this section (OPSA has a long response time for uploading the emergency flow information and downloading the optimal path calculated by the network controller; this response time is much longer than a regular flow’s transmission time). Some simulation parameters are summarized in Table 1. In considering the features of industrial networks, our experiment runs under a multi-channel TDMA protocol, and the number of nodes in the network is less than 100. The number of channels in the simulations is m=16; without loss of generality, the variation range of the number of nodes is from 30–80; based on the number of nodes, the number of flows in our experiment is from 13–18.

To illustrate the applicability of our approach, several test cases were randomly generated for each parameter configuration. The network gateway is placed in the center of playground area *A*, and the other sensors are deployed around the gateway randomly. When setting the transmission range as d=40 m, the number of nodes *n* and the playground area *A* should satisfy [30]:

(13) $$\frac{n}{A}=\frac{2\pi }{d^{2}\sqrt{27}}.$$

Two nodes can communicate with each other when the distance between each other is less than *d*; they are adjacent nodes. We can obtain the network topology by randomly connecting the nodes from each source node to the destination. If some source nodes cannot be connected to the destination, their locations are randomly generated again. Notably, the paths in typical industrial networks are connected randomly and are not the shortest paths [13,27].

In order to control the workload of the entire network, we use the utilization *u* in our simulations. The network utilization is specified to obtain flow sets, $U=\sum u_{i}$(U<1), and the UUniFastalgorithm [31] is used to generate each flow’s utilization, $u_{i}$ ($u_{i}=\frac{c_{i}}{t_{i}}$). The result generated by the UUniFast algorithm follows a uniform distribution, which is neither pessimistic nor optimistic for the analysis [31].

**Figure 8:**
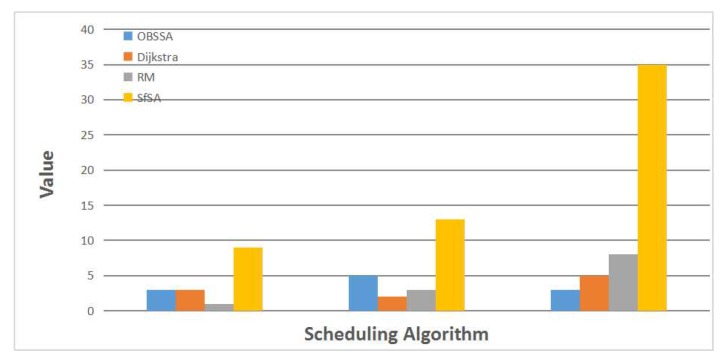
An example of one test case.

Figure 8 shows an example of one test case. In this example, in accordance with the actual situation, we set the number of nodes to n=50, the number of flows to F=15 and the network utilization to U=0.3. The deadline for the emergency flow in this case is four. The three sets of values on the x axis, from left to right, represent the number of stolen flows, the transmission path length and the actual number of transmission slots. From the first set of values, we can find that the traditional RM scheme needs to steal resources from only one flow for transmission, whereas both OBSSA and the Dijkstra algorithm steal from three flows. SfSA, in this case, needs to steal from nine flows to transmit the emergency packet to the destination. The Dijkstra algorithm uses only two hops because it is a shortest path algorithm. Obviously, the lengths of the transmission paths for the different algorithms satisfy SfSA>OBSSA>RM>Dijkstra. However, only OBSSA can meet the deadline of the emergency flow, using 3<4 slots. This is because OBSSA dynamically selects the transmission path at each intersection depending on current conditions. When the packet can be scheduled on the current path or on the path of a flow from which resources have already been stolen, it will stop stealing from new flows. In this case, when the deadline of the emergency flow is eight, OBSSA will degrade into the RM scheme.

**Figure 9:**
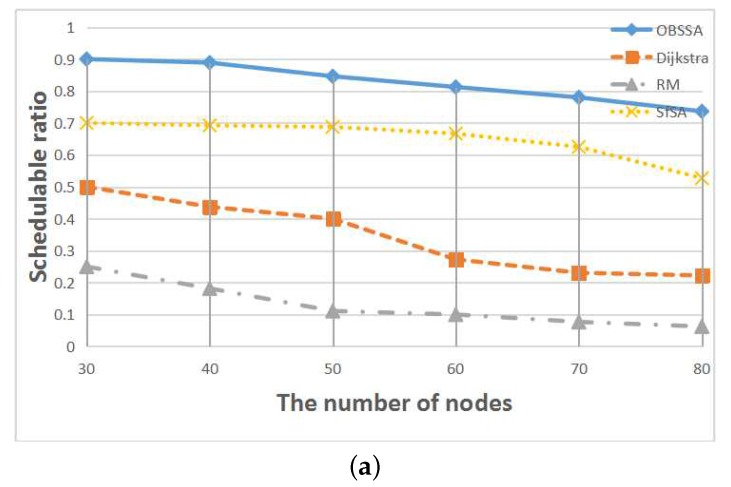

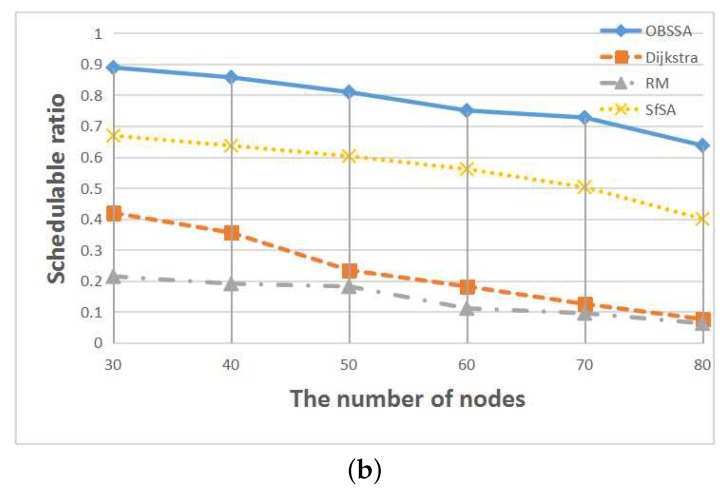
The relationship between the schedulability ratio and the number of nodes. (**a**) F=15, U=0.4; (**b**) F=15, U=0.5.

To compare the performances of these scheduling algorithms, we now consider test cases of varying sizes. Figure 9 shows the relationship between the schedulability ratio and the number of nodes for F=15 flows and network utilizations of U=0.4 and U=0.5. Numbers of nodes between N=30 and N=80 are considered. This node scale is realistic because in typical industrial WSNs, a WirelessHART network with one gateway comprises 50–80 field devices. Figure 9 illustrates that the schedulability ratios of all scheduling algorithms decrease with an increasing number of nodes. However, our scheduling algorithm OBSSA can maintain a high schedulability ratio at the cost of a small amount of storage space. This is because the emergency packet can always choose an optimal path at each intersection to guarantee or improve its timely forwarding to its destination. As the number of nodes increases, the number of intersections decreases. Hence, the schedulability ratio of OBSSA declines. When the system storage space is limited and is not enough to store intersection information, the system can use SfSA as an alternative means of achieving an acceptable schedulability ratio. This is because in a system that uses the RM strategy, a flow with a smaller period always has a higher priority. Therefore, the emergency packet can arrive at its destination within a short time. However, the packet may miss its deadline in some situations when it does not select the most direct path, as shown by the example in Figure 4. The schedulability ratios of the traditional RM scheme and the Dijkstra algorithm are lower than those of OBSSA and SfSA. Furthermore, these two methods suffer faster performance degradation. The reason is that neither the RM scheme nor the Dijkstra algorithm consider the time factor for the emergency flow when choosing paths. Hence, neither can achieve a high schedulability ratio. When the network utilization is increased from U=0.4 to U=0.5, the schedulability ratios of all scheduling algorithms decrease more rapidly. However, the schedulability ratio of OBSSA remains at an acceptable level.

**Figure 10:**
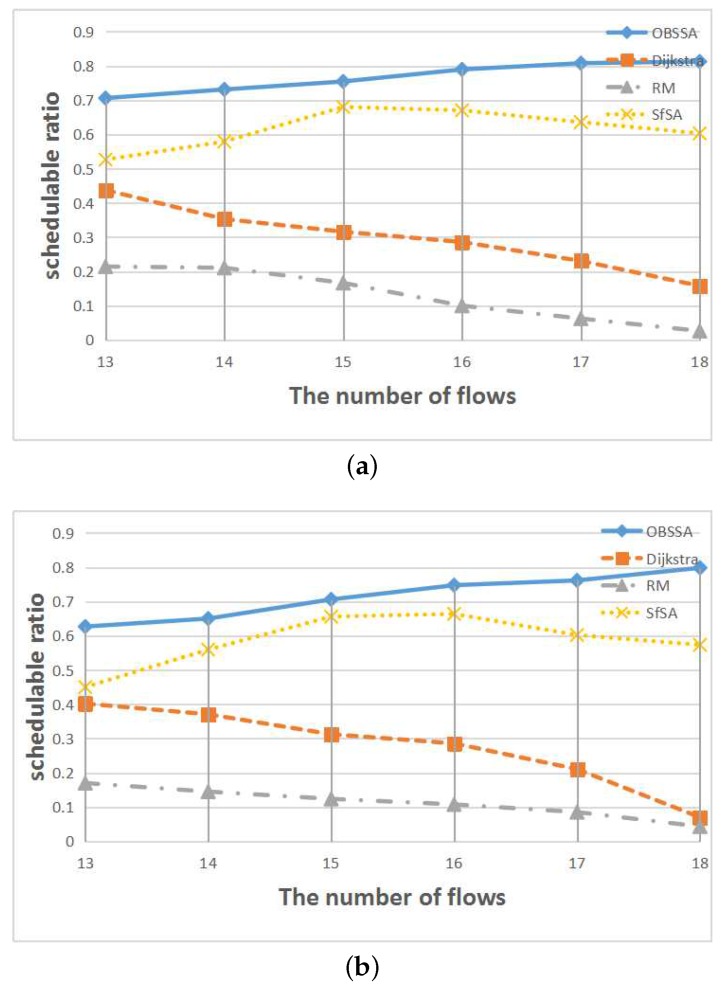
The relationship between the schedulability ratio and the number of flows. (**a**) N=50, U=0.4; (**b**) N=50, U=0.5.

Figure 10 shows the relationship between the schedulability ratio and the number of flows for N=50 nodes and network utilizations of U=0.4 and U=0.5. Numbers of flows between F=13 and F=18 are considered. The results are similar to those presented in Figure 9. The schedulability ratios of both the RM scheme and the Dijkstra algorithm are continuously decreasing. By contrast, the schedulability ratio of OBSSA increases with an increasing number of flows, whereas the schedulability ratio of SfSA initially increases and then begins to decrease when the number of flows reaches approximately 15 or 16 (since the test cases were generated randomly, the peak values are not the same between the two utilization cases). The reason is that as the number of flows increases, the number of intersection nodes also increases, which increases the number of links available to transmit emergency packets. Hence, the schedulability ratio of OBSSA increases with an increasing number of flows. As the number of flows continues to increase, more packets must be transmitted via long detours. Thus, the schedulability ratio of SfSA begins to trend downward.

**Figure 11:**
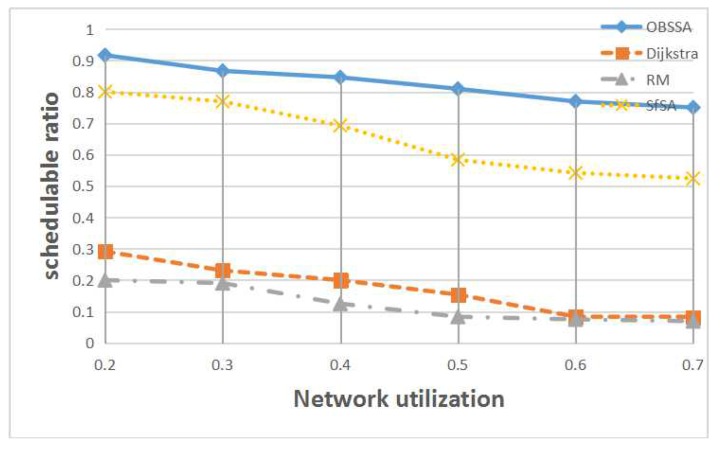
The relationship between the schedulability ratio and the network utilization.

Figure 11 shows the relationship between the schedulability ratio and the network utilization for F=15 flows and N=70 nodes. Levels of network utilization between U=0.2 and U=0.7 are considered. Obviously, OBSSA has the highest schedulability ratio for each level of network utilization. This is because for a given network topology and routing, the controller can abstract the transmission information of the intersections as shown in Figure 7. With this information downloaded to each intersection, OBSSA can dynamically choose the optimal path. However, the schedulability of the system decreases with increasing network utilization. When there is no path that can reach the destination before the deadline of the emergency flow or when the source of the emergency flow needs a long time to reach the optimal path, the schedulability ratio of OBSSA degrades. Additionally, the schedulability ratios of the Dijkstra algorithm and the RM scheme are very low; emergency flows are difficult to schedule when U>0.4 (the schedulability ratio is lower than 0.2).

**Figure 12:**
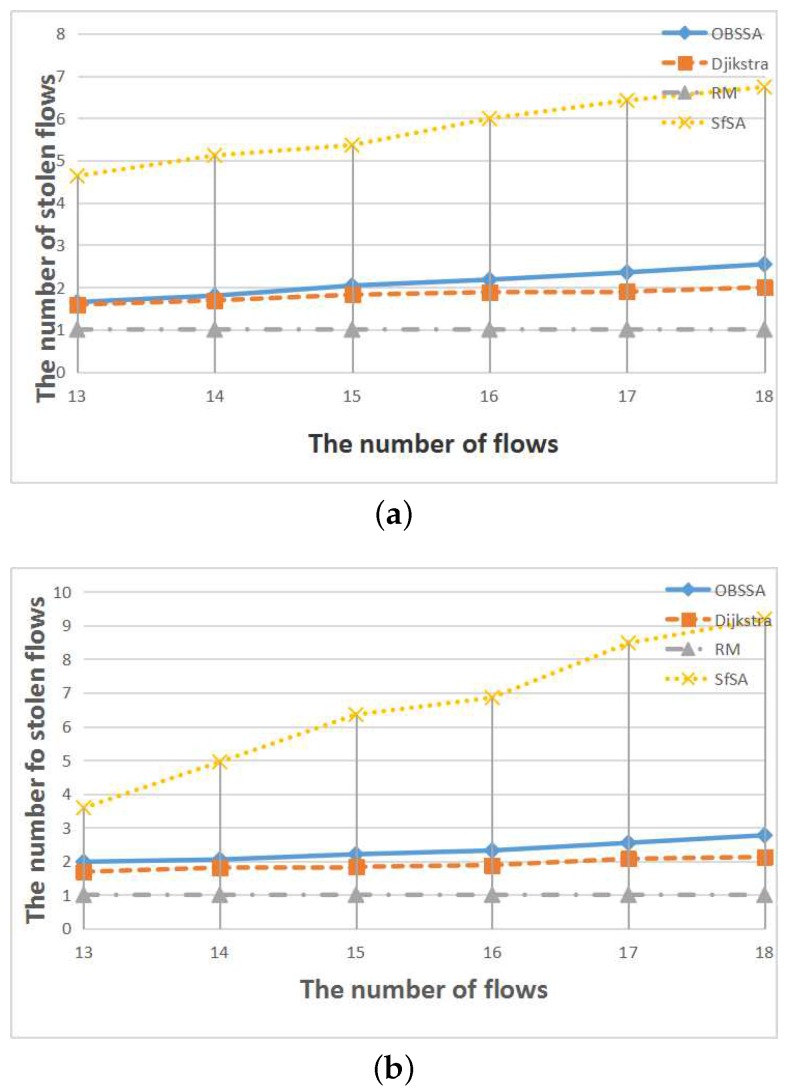
The relationships between the number of stolen flows and the number of nodes. (**a**) N=50, U=0.4; (**b**) N=50, U=0.5.

**Figure 13:**
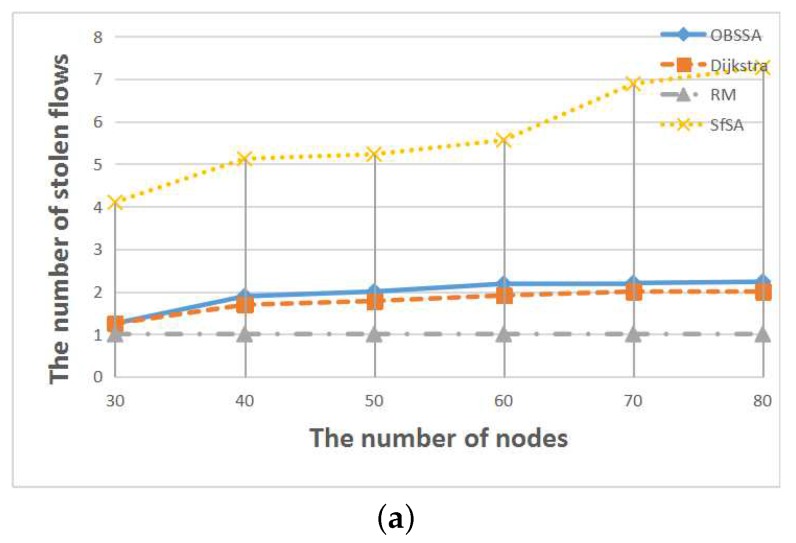

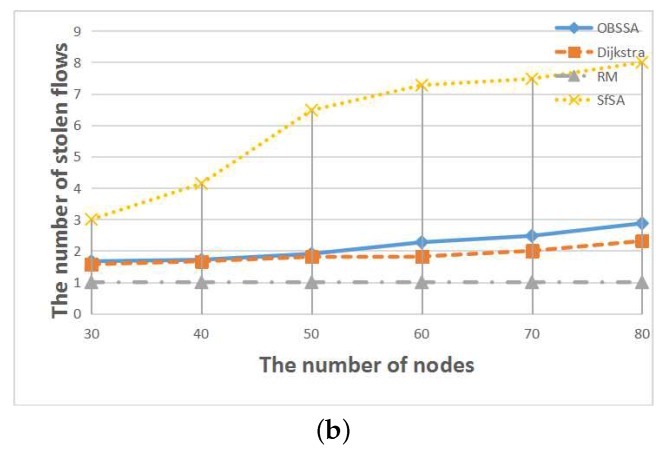
The relationships between the number of stolen flows and the number of flows. (**a**) F=15, U=0.4; (**b**) F=15, U=0.5.

**Figure 14:**
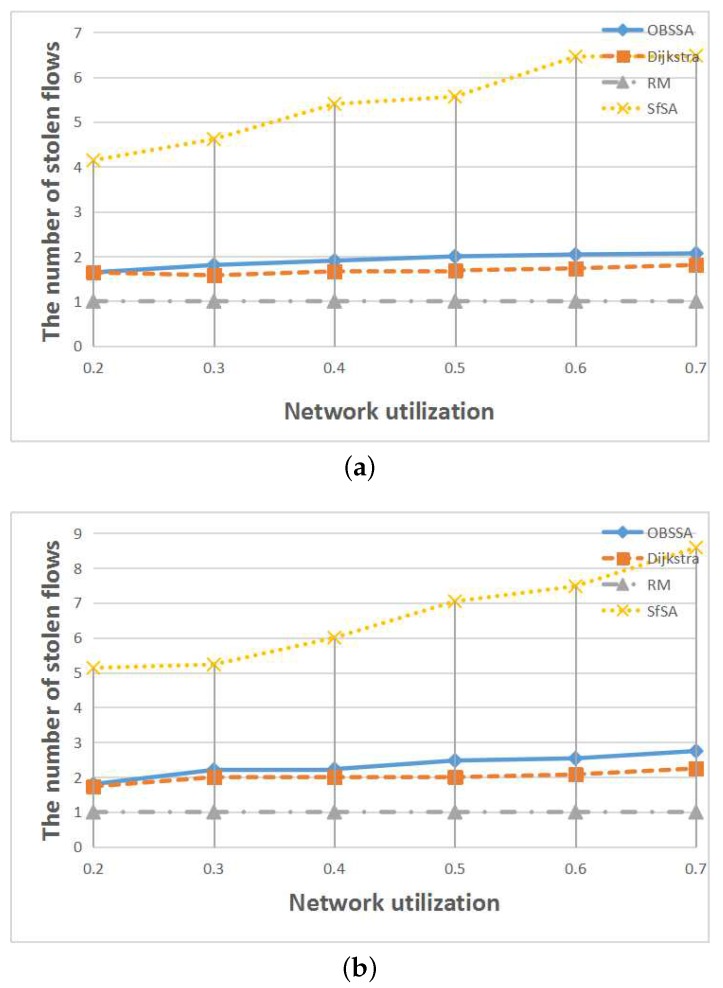
The relationships between the number of stolen flows and the network utilization. (**a**) N=50, F=15; (**b**) N=70, F=15.

To compare the number of stolen flows when an emergency flow is scheduled for each scheduling algorithm, we extended the deadline of the emergency flow to each algorithm’s arrival time in each test case. This is also a very important consideration for real-world applications because regular flows can provide assistance in analyzing an accident. Figure 12, Figure 13 and Figure 14 show the relationships between the number of stolen flows and the network parameters, namely the number of nodes, the number of flows and the network utilization. Since SfSA is a locally optimal algorithm and needs to steal resources from many flows to reduce the waiting time regardless of whether the current path permits arrival at the destination in time, SfSA has the largest number of stolen flows. By contrast, the RM scheme steals only one path because it transmits only on the originally-chosen path. The numbers of stolen flows under OBSSA and the Dijkstra algorithm are always similar and much smaller than the number for SfSA. This is because when the emergency flow can be scheduled, OBSSA stops stealing new flows. Hence, the number of stolen flows in OBSSA increases only slowly. This shows that OBSSA not only has a higher schedulability ratio, but also a lesser impact on regular flows.

## 7. Conclusions

Reliability and real-time performance are the most important characteristics of industrial WSNs. By means of centralized control, WSNs can provide real-time performance and reliability in industrial production. In standards such as WirelessHART, reliable graph routing approaches are adopted to improve the reliability of industrial system. However, such a centralized controller cannot effectively respond to emergencies, and emergency packets may miss their deadlines. To address this issue, we improve the reliability of scheduling for emergency tasks by proposing a method based on a stealing mechanism. In our method, an emergency task is transmitted by stealing resources allocated to regular flows. The challenges addressed in our work are as follows: (1) emergencies occur only occasionally, but the industrial system must deliver the corresponding flows within their deadlines when they occur; and (2) we wish to minimize the impact of emergency flows by reducing the number of stolen flows. The contributions of this work are as follows:

1. We propose an optimal algorithm for emergency flows (OPSA); by iterating over all scenarios, the controller can determine the optimal path for an emergency flow. However, because the network is centrally controlled, the source node of the emergency flow needs to upload its information and wait for a response from the network controller. The real-time performance of OPSA is poor. Then, we propose a series of algorithms (such as SfSA, LSSA) to try to improve system performance.
2. We define intersection (such as the intersection nodes or overlap nodes of several flows) and blocking (such as the delay caused by insufficient transmission resources) as new characteristics of flows.
3. We prove that the shortest path is not always the fastest path for an emergency flow triggered by an unpredictable accident.
4. We analyze the transmission time from $B_{0}$ to the intersection node on the path of flow *i* and obtain the optimal path as the one that satisfies Equation (10) with the minimum number of intersections in $P_{is}$.
5. We propose an algorithm (OBSSA) that can determine optimal paths for emergency flows. The optimal path in the current study is the path that allows an emergency task to arrive at its destination before its deadline with the minimal impact. Furthermore, we prove that the performance of OBSSA is no less than OPSA when we do not consider the overhead of uploading and downloading.

We demonstrate that our method and analysis approach significantly outperform existing ones by extensive simulations.

Future work will further analyze the relationship between the behavior of OBSSA/SfSA and the number of flows. Since the performance of OBSSA under different scheduling policies (Earliest Deadline First, Rate Monotonic, Fixed Priority, and so on) is different, we will also seek to study how to improve reliability by optimizing the scheduling policy based on OBSSA for industrial WSNs.

## Figures and Tables

**Table 1:** Simulation parameters.

Parameter	Description
*n*	the number of nodes
*d*	transmission range
*U*	network utilization
$u_{i}$	flow i ’s utilization
*F*	the number of flows
